# Scleredema of Buschke: 2 cases with a remarkable response to treatment of the underlying paraprotein

**DOI:** 10.1016/j.jdcr.2025.12.033

**Published:** 2025-12-27

**Authors:** Evangelia Vetsiou, Laura Adams, Marianna Philippidou, Rabi Nambi, Kirsty Cuthill, Tanya N. Basu

**Affiliations:** a Department of Dermatology, King’s College Hospital, London, United Kingdom; b Department of Dermatology, University Hospitals of Derby and Burton, Derby, United Kingdom; c Department of Histopathology, King's College Hospital, London, United Kingdom; d Department of Haematology, King's College Hospital, London, United Kingdom

**Keywords:** bortezomib, Buschke, MGCS, monoclonal gammopathy of cutaneous significance, paraprotein, scleredema

## Introduction

Type II scleredema of Buschke is a monoclonal gammopathy of cutaneous significance, in which progressive skin changes appear driven by low-level serum paraprotein. We report 2 cases of debilitating, treatment-refractory scleredema worsening over the years, with marked remission following paraprotein-directed therapy.

## Case report

Two female patients, aged 59 and 69, presented with a 2-year history of progressive cutaneous tightening involving the face, neck, and upper torso. Both patients had limited mouth opening, difficulty chewing food, restricted shoulder motion, and periorbital swelling, leading to significant functional impairment. One required a wheelchair; the other ceased golf and horse riding, becoming largely housebound.

**Fig 1:**
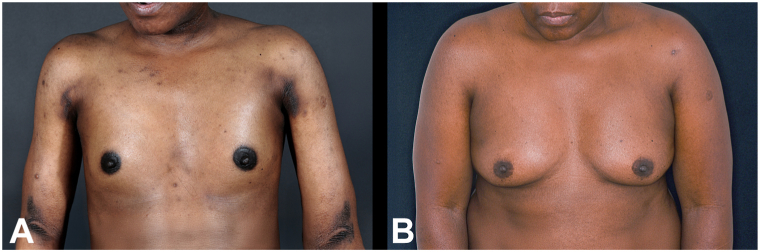
The woody induration encasing the torso has caused flattening of the breast tissue **(A)**. Remarkably, this has now fully resolved following plasma cell-directed therapy, allowing reappearance of the normal breast contour **(B)**.

**Fig 2:**
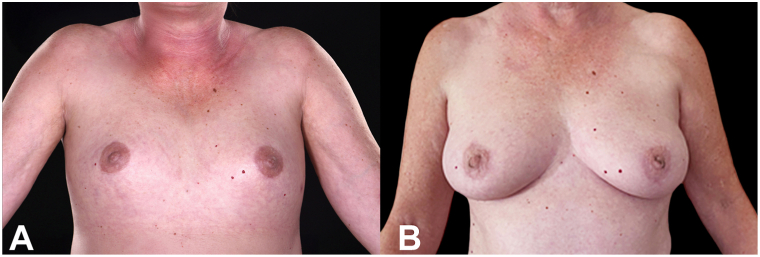
Similarly, in patient 2, flattening of the breast tissue is observed due to woody induration encasing the torso **(A)**. This has fully resolved following plasma cell-directed therapy, and a normal breast contour is now evident **(B)**.

Examination revealed diffuse, woody skin induration with overlying rippled hyperpigmentation (Figs 1, *A* and 2, *A*). Autoantibodies (antinuclear antibody, extractable nuclear antigen, and Scl-70), haemoglobin A1c, serum glucose, and anti-streptolysin O, were unremarkable in both patients.

Serum protein electrophoresis revealed an IgA κ paraprotein (11 g/L) in the first patient and an IgG λ paraprotein (11 g/L) in the second patient. Bone-marrow biopsy demonstrated distinct pathological profiles; 20% to 30% clonal plasma cells in the first patient confirmed multiple myeloma. The second patient exhibited approximately 10% clonal plasma cells with λ light chain restriction, consistent with a diagnosis of myeloma. These findings meet the International Myeloma Working Group criteria for the diagnosis of multiple myeloma, but neither patient met the hematological criteria for myeloma treatment.

**Fig 3:**
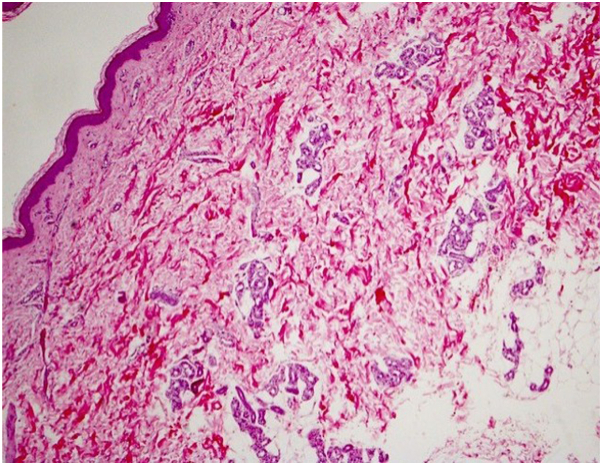
The epidermis is generally unaffected with mild atrophy. There is an expansion of the reticular dermis, with collagen extending also into the subcutis, resulting in an apparently abnormal placement of the eccrine coils. (Hematoxylin-eosin stain; original magnification:×10.)

**Fig 4:**
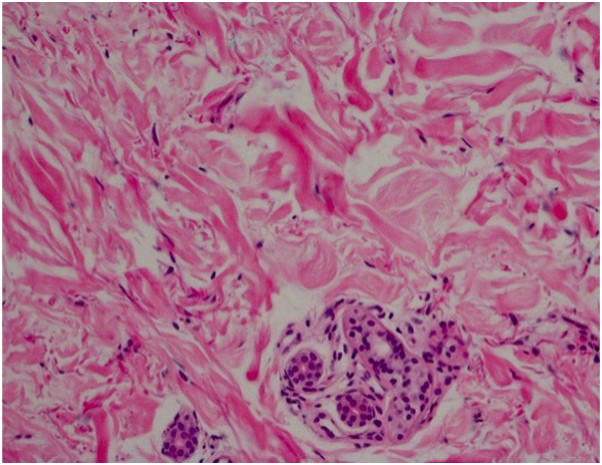
The collagen fibers are swollen and separated from one another. The extent of this separation mirrors the amount of interstitial mucopolysaccharide present and depends on the stage of the disease. In addition, the eccrine coils have lost periadnexal fat. (Hematoxylin-eosin stain; original magnification:×40.)

**Fig 5:**
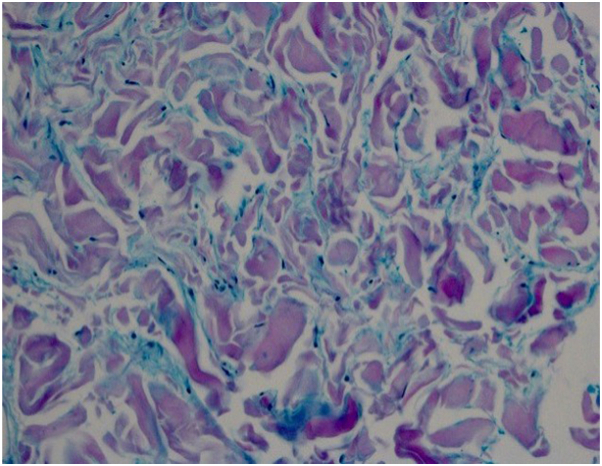
Special stain highlighting the deposition in blue of interstitial mucopolysaccharide. (Alcian blue stain; original magnification:×40.)

Skin biopsies in both cases demonstrated reticular dermal expansion, separation of hyalinized collagen bundles (Figs 3 and 4), and interstitial mucin on Alcian blue staining (Fig 5), confirming scleredema of Buschke.

Over several years, both cases remained refractory to various treatments, including UV-A1, mycophenolate-mofetil, and intravenous immunoglobulin. Because of progressive, debilitating symptoms, a multidisciplinary consensus was reached to offer paraprotein-directed therapy to treat the scleredema, recognized as a monoclonal gammopathy of clinical or cutaneous significance.^1^

Consequently, the first patient was started on a bortezomib-based regimen (bortezomib, cyclophosphamide, and dexamethasone). The patient received 5 cycles at 3-week (21-day) intervals. The regimen comprised subcutaneous bortezomib at 1.3 mg/m^2^, oral cyclophosphamide at 500 mg weekly, and dexamethasone at 20 mg weekly. A marked improvement in skin thickening and mobility was observed. Quality of life improved dramatically, as measured by the Dermatology Life Quality Index, which decreased from 23/30 to 1/30. Treatment response was also assessed using serial US to measure skin thickness at fixed anatomical points and the modified Rodnan skin score (typically used to assess scleroderma), which improved from 32% to 14% immobile areas.

Similarly, after failure of conventional therapies, the second patient was commenced on a bortezomib-based regimen (daratumumab, bortezomib, thalidomide, and dexamethasone). The patient received 6 cycles comprising weekly subcutaneous bortezomib (1.3 mg/m^2^), subcutaneous daratumumab (1800 mg weekly), and dexamethasone (20 mg weekly), followed by 2 additional cycles of bortezomib alone administered fortnightly. An equally striking clinical response was noted. Thalidomide was omitted due to dysphagia.

Both patients demonstrated a strong biochemical response to treatment. In particular, in patient 1, the IgA κ paraprotein level declined from 11 to 2 g/L (representing a >50% reduction), with this response sustained for 2 years. A mild and gradual increase has been observed since, with current levels stabilizing between 7 and 8 g/L, 8 years post-treatment, and without the emergence of new symptoms or clinical manifestations.

In patient 2, the IgG λ paraprotein level decreased from 11 g/L to nearly undetectable levels (>90% reduction), with the response maintained to date, 2 years following completion of therapy.

Both patients now have almost complete resolution of scleredema. Skin examination is normal with good mobility (Figs 1, *B* and 2, *B*). Our first patient no longer uses a wheelchair and can travel again, whereas the second can again enjoy golf and horse riding. They remain under 6-monthly surveillance by our hematology colleagues with no requirement for further treatment.

## Discussion

Both our patients had type 2 scleredema of Bushke, a rare, progressive mucinous connective tissue disorder associated with an underlying monoclonal gammopathy. Scleredema may also be associated with infection (type 1, usually Streptococcal) and diabetes (type 3).^2^ It presents with symmetrical, woody induration of skin on the upper body, progressing distally but typically sparing the hands and feet. Although usually confined to the skin, ocular (muscle palsies), gastrointestinal (dysphagia), and respiratory (dysphonia) involvement have been reported. Clinically, differentiation from scleroderma, eosinophilic fasciitis, and scleromyxedema is essential (the latter is also associated with monoclonal gammopathy and increased intradermal mucin on histopathology, see Table I).

**Table I:** Differentiating sclerotic skin conditions: key diagnostic investigations for scleredema of Buschke, scleroderma, eosinophilic fasciitis, and scleromyxedema

Condition	Paraprotein association	Other diagnostic features and investigations	Histologic features
Scleredema of Buschke	• Often associated with monoclonal gammopathy, particularly IgG κ type • Found in ∼25% of cases	• May be preceded by Streptococcal infection or diabetes • Magnetic resonance imaging/ultrasound: thickening of dermis/subcutaneous tissue	• Affects the reticular dermis comprising thickened swollen collagen fibers, minimal inflammation but striking deposition of dermal mucin
Scleroderma	• Not typically associated with paraproteinemia	• Capillaroscopy: nailfold capillary changes • Autoantibodies: antinuclear antibody (usually positive), anti-Scl-70 (diffuse), anti-centromere (limited)	• Affects both the dermis and subcutis showing dense hyalinized collagen, perivascular inflammation, and minimal mucin deposition
Eosinophilic fasciitis	• Not commonly associated with paraproteinemia	• Peripheral eosinophilia (50%-60% of cases) • Magnetic resonance imaging: fascia thickening • Deep biopsy: confirms fascia involvement	• Affects the deep fascia ± muscle with only secondary involvement of dermal collagen and with eosinophil-rich inflammation. No dermal mucin deposition
Scleromyxedema	• Almost always associated with monoclonal gammopathy (IgG λ)	• Systemic involvement: neuropathy, renal, pulmonary, and gastrointestingal involvement	• Affects the dermis with expanded and disorganized collagen, variable inflammation marked mucin deposition, and a conspicuous increase in dermal fibroblasts

In type 2 scleredema of Buschke, the mechanism by which paraproteins drive disease remains unclear. Ohta et al^3^ showed that affected patients’ serum stimulates collagen production in skin fibroblast cultures, implicating circulating paraprotein-related factors in promoting extracellular matrix synthesis and dermal fibrosis. Others hypothesize that paraproteins directly activate fibroblasts^4^ or interact with other connective tissue antigens.^5^

Assessing disease response to treatment is modified Rodnan skin score challenging. We found serial ultrasonography aided disease evaluation, objectively measuring skin thickness at fixed points over time. Although the is not validated in scleredema, this was also helpful. Grudeva-Popova and Dobrev proposed noninvasive skin elasticity measurements as another useful method for evaluating treatment response.^1^

Management of type 2 scleredema of Buschke is difficult; narrowband UV-B phototherapy and intravenous immunoglobulin can yield some benefit. Other reported therapies include electron beam therapy and extracorporeal photopheresis with varying success rates.^6^, ^7^, ^8^ The importance of physiotherapy and psychological support can be underestimated.

Initial attempts at paraprotein-directed therapy for type 2 scleredema of Buschke did not always give clinical improvement (regimens included melphalan, cyclophosphamide, vincristine, and thalidomide with steroids).^9^ Therapies such as bortezomib, a proteasome inhibitor, have the potential to reduce paraprotein levels to undetectable levels.^6^^,^^10^ We speculate that this is particularly important in the management of monoclonal gammopathies of cutaneous significance, where even minimal amounts of paraprotein can drive skin disease. Notably, our second patient was treated with a combination of bortezomib, daratumumab, and dexamethasone. This is a key distinction because daratumumab—a highly effective anti-CD38 monoclonal antibody—was not available at the time the first patient was treated. Currently, daratumumab is not funded for patients with <10% clonal plasma cells because they fall short of the diagnostic threshold for myeloma. Collecting evidence to support its efficacy in such cases of monoclonal gammopathy of clinical or cutaneous significance could help inform efforts to revise existing funding criteria and expand access to this potentially transformative treatment.

In summary, recognizing type 2 scleredema of Buschke as a monoclonal gammopathy of clinical (or cutaneous) significance can enable life-changing treatment. We were struck by how much of the longstanding scleredema was reversible with treatment. Dermatologists should advocate for their patients in cases of monoclonal gammopathy of clinical or cutaneous significance. We highlight that treatment of the underlying monoclonal gammopathy can be extremely helpful to treat this progressive, incapacitating condition, even when patients do not fulfill the hematological criteria for treatment.

## Conflicts of interest

None disclosed.
